# Visual search in ADHD, ASD and ASD + ADHD: overlapping or dissociating disorders?

**DOI:** 10.1007/s00787-020-01535-2

**Published:** 2020-04-20

**Authors:** D. Seernani, K. Damania, C. Ioannou, N. Penkalla, H. Hill, T. Foulsham, A. Kingstone, N. Anderson, G. Boccignone, S. Bender, N. Smyrnis, M. Biscaldi, U. Ebner-Priemer, Christoph Klein

**Affiliations:** 1 Department of Child and Adolescent Psychiatry, Medical Faculty, University of Freiburg, Hauptstrasse 8, 79104 Freiburg, Germany; 2 Seattle, USA; 3 Institute of Sports and Sports Sciences, Karlsruhe Institute of Technology, Karlsruhe, Germany; 4 Department of Psychology, University of Essex, Colchester, UK; 5 Department of Computer Science, University of Milan, Milan, Italy; 6 Brain, Attention and Reality Lab, University of British Columbia, Vancouver, Canada; 7 Department of Child and Adolescent Psychiatry, Medical Faculty, University of Cologne, Cologne, Germany; 8 Department of Psychiatry, National and Kapodistrian University of Athens, Athens, Greece

**Keywords:** Attention-deficit/hyperactivity disorder (ADHD), Autism spectrum disorder (ASD), Local processing, Intra-subject variability (ISV), Visual search

## Abstract

Recent debates in the literature discuss commonalities between Attention-Deficit/Hyperactivity Disorder (ADHD) and Autism Spectrum Disorder (ASD) at multiple levels of putative causal networks. This debate requires systematic comparisons between these disorders that have been studied in isolation in the past, employing potential markers of each disorder to be investigated in tandem. The present study, choose superior local processing, typical to ASD, and increased Intra-Subject Variability (ISV), typical to ADHD, for a head-to-head comparison of the two disorders, while also considering the comorbid cases. It directly examined groups of participants aged 10–13 years with ADHD, ASD with (ASD+) or without (ASD−) comorbid ADHD and a typically developing (TD) group (total *N* = 85). A visual search task consisting of an array of paired words was designed. The participants needed to find the specific pair of words, where the first word in the pair was the cue word. This visual search task was selected to compare these groups on overall search performance and trial-to-trial variability of search performance (i.e., ISV). Additionally, scanpath analysis was also carried out using Recurrence Quantification Analysis (RQA) and the Multi-Match Model. Results show that only the ASD− group exhibited superior search performance; whereas, only the groups with ADHD symptoms showed increased ISV. These findings point towards a double dissociation between ASD and ADHD, and argue against an overlap between ASD and ADHD.

## Introduction

Autism Spectrum Disorder (ASD) is characterised by impairments in social communication and social interaction, and repetitive or restrictive behaviour and activities; and Attention-Deficit/Hyperactivity Disorder (ADHD) by inattention, hyperactivity and impulsivity [4]. Up until 2013, the DSM-IVR diagnostic criteria considered ADHD-like symptoms in ASD to be symptomatic phenocopies and attributed these to the ASD diagnosis. The 5th Diagnostic and Statistical Manual [4] for the first time allowed the co-morbid diagnosis of ASD with ADHD. This new classification proved reasonable as the estimated comorbidity rates of ADHD and ASD have ranged at high levels from 37 to 78% [51]. The prevalence of these comorbidities seems to increase during childhood and peak around adolescence [24]. Both disorders are highly heritable with > 90% heritability estimated for ASD and 70–76% estimated for ADHD [17, 46].

Some research groups have tried deciphering the shared and common etiological factors of ADHD and ASD by studying the two disorders in tandem, with or without a co-morbid group. These studies have measured a variety of constructs and functions, for instance, face recognition, face and gaze direction, imitation, motor function, working memory, attention, inhibition, executive function and intra-subject variability [7, 48, 50, 52, 53]. While atypical performance on some of these constructs has been common to both disorders, most others show clear dissociation; with the ASD + ADHD group occasionally showing additive effects of both disorders.

### Performance on the visual search task

ASD is not only characterised by deficits, such as those reported above, but also is associated with superior performance in several tasks requiring heightened local processing as in the Hidden Pictures and Embedded Figures Tests [2, 3, 25, 31, 42]. These findings have been explained by different theories, such as weak central coherence [11] and enhanced perceptual functioning [39]. Both these models describe ASD groups as having better performance on locally oriented tasks—that is, tasks where global stimuli need to be evaluated with regard to one or more individual local entities. An example of such a task is the visual search task where the participant is required to find a target object (e.g., a blue circle) amidst a number of distracting objects (e.g., different coloured shapes such as blue squares and circles).

A large number of studies have shown superior performance of ASD on visual search tasks, as indicated by shorter reaction times (RTs), as well as fewer and shorter gaze fixations [25, 27, 31, 43]. However, other investigations [21, 26, 37] have failed to observe this advantage. In addition to power issues and lack of task standardisation, a potential cause of this discrepancy is the presence of clinical comorbidities along with ASD. None of the visual search studies mentioned above report the presence of a comorbid ADHD group or stimulant medications taken by participants, within their larger ASD samples. To our knowledge, only one study [15] investigated visual search in Autism with a focus on comorbid ADHD symptoms, reporting that the group of 3-year olds with comorbid ASD + ADHD symptoms showed significant less spatially systematic search than the “purely” autistic group and, thus, showed worse performance.

While the visual search task has been investigated thoroughly in ASD research, only a limited number of studies have looked at visual search performance in ADHD. Mullane and Klein [40] provide a literature review of these few studies and complementing these with a qualitative analysis. These investigators reported that ADHD children are less efficient at search only at the lowest or highest level of complexity, possibly due to boredom or inefficient resource allocation, respectively.

### Intra-subject variability

Increased reaction-time variability is one of the most replicable findings in the ADHD literature [28, 33–35, 48]. This within-subject moment-to-moment fluctuation of task performance is known as Intra-Subject Variability (ISV). The few studies that measured ISV in an ASD group compared to an ADHD group have yielded mixed results [19, 55]. However, studies that directly compared ASD− (no comorbid ADHD) and ASD+ (ASD with comorbid ADHD) groups with an ADHD group have found evidence of increased ISV in ASD only in the presence of comorbid ADHD [48, 52, 53].

While ISV of manual reaction times has been well documented, only a few studies have looked at ISV in oculomotor measures [29, 30, 38, 41], each of these studies reporting increased oculomotor ISV in ADHD as compared to TD groups, and none of them directly comparing ADHD with “purely” autistic or comorbid patients.

### The role of potential endophenotypes

A useful approach to studying possible etiological overlaps and dissociations of underlying psychopathologies, such as those of ASD and ADHD, is to search for endophenotypes. Endophenotypes are defined as measurable variables along the pathway between genotype and observable disease [22]. These variables could be neurophysiological, biochemical, endocrinological, neuroanatomical, cognitive or neuropsychological. To qualify as an endophenotype, the variable must be associated with the illness in the population, be heritable, state independent, co-segregate within families, and be present in nonaffected family members at a higher rate than the general population [22].

ISV is a candidate endophenotype of ADHD [28, 33–35, 45]. There is some evidence to suggest that heightened performance on tasks like visual search is specific to ASD [25, 27, 31, 42, 43], state independent, present before the manifestation of the ASD phenotype [12, 20] and is present in non-affected family members [9]. Thus, superior visual search performance may also be a candidate endophenotype of ASD.

### Study goals

Based on the above evidence, the present study aimed to examine (1) performance in visual search and (2) intra-subject variability, simultaneously in TD, ADHD, ASD− and ASD+ groups. To effectively analyse the process of carrying out a visual search task in the typical and clinical groups, oculomotor parameters will be analysed [32]. Potential underlying etiological overlaps between ADHD and ASD are expected to manifest as increased ISV and at par (or statistically not different from each other) performance on the visual search task in all three clinical groups. A double dissociation between ASD and ADHD will be manifested with increased ISV in the ADHD group and superior performance in visual search in the ASD− group. Based on the present literature, it is difficult to predict, a priori, what pattern the ASD+ group will manifest with respect to performance of visual search and ISV. It is possible that the ASD+ group follow the ASD− trends alone showing superior performance at search and no increased ISV, or vice versa with increased ISV but no superior search or manifest an addition of ASD and ADHD.

## Methods

The present study has been approved by and conducted in accordance with the ethics committee of the University of Freiburg.

### Participants

For the present study, *N* = 100 participants aged 10–13 years were recruited across four groups—typically developing (TD) participants, participants with Attention-Deficit/Hyperactivity Disorder (ADHD), Autism Spectrum Disorder without co-morbid ADHD (ASD−), and Autism Spectrum Disorder with co-morbid ADHD (ASD+) (see Table 1). Of these, 3 participants were excluded because of incomplete testing sessions, 3 for IQ lower than 70 points, 2 for a reading disorder diagnosis, 3 for unverifiable diagnosis, and 4 for poor data quality (e.g. unreliable oculomotor data due to poor calibration, failure to accurately calibrate, multiple responses on over 50% of trials, button presses before and during the start of search rendering accurate segmentation impossible, etc.). Two of the participants from the ADHD group could not participate in the IQ testing session. These participants were included based on previous records of IQ tests using the Wechsler Intelligence Scale for Children and after consulting with the clinicians in charge, but they were excluded from IQ correlations and ANCOVAs using IQ as a covariate. In sum, data of *N* = 85 participants were included for analysis. All participants in the clinical groups were diagnosed by and recruited through the Department of Child and Adolescent Psychiatry, University Medical Centre Freiburg. All diagnoses were made by experienced clinicians according to the ICD-10 Criteria. ADHD diagnosis was based on interviews with parents and children, behavioural observations and the German version of the Conner’s parent and teacher rating scale. ASD Diagnosis was based on the Autistic Diagnosis Observation Schedule and Autism Diagnostic Interview-Revised, the gold standards for Autism Diagnosis. The TD participants were recruited through the departmental database, including data of participants in local schools and sports groups interested in participating in studies, and by advertising through employees of the University Hospital Freiburg. A telephonic conversation with the parents was used to confirm that the participants in the TD group had no known psychiatric or neurological history. In the three clinical groups, there were 7 participants with Enuresis, 6 with Adjustment Disorder, 1 with Social Phobia, 1 with Specific Phobias, 1 with Childhood Emotional Disorder, 1 with Depressive Episodes, 1 with Dyscalculia, 1 with Specific Spelling Disorder, 1 with Developmental Dyspraxia, 4 with Expressive Language Disorder, 2 with Tic Disorder, 1 with Somnambulism, 1 with Obsessive–Compulsive Disorder. Parents of all participants filled out the Child Behavior Checklist (CBCL; [1]) and Social Responsiveness Scale (SRS; [10]) as a control measure for general psychological asymptomatology and autism symptoms respectively. In order to assess ADHD symptoms, all three clinical groups were given the German language questionnaires ‘Diagnostik-Systeme für Psychische Störungen im Kindes- und Jugendalter’ (DISYPS) (ADHD-FBB reported by parents and ADHD-SBB which is a self-report scale; [16]). Participants were requested to not take stimulant medication on the day of testing. The four groups were matched in age, and no significant between-group differences were found on IQ (as measured by the Culture Fair Intelligence Test 20 (CFT 20-R; [56]) and gender. Detailed demographics can be found in Table 1. The questionnaires were collected as supplementary tools by researchers to complement group diagnosis. Further, these were occasionally used in individual cases where clinicians could provide insights about a participant’s group membership. This, along with the clinical expertise provided by the clinicians in-charge, was particularly useful to distinguish the ASD+ and ASD− group membership for those participants who were diagnosed and treated before DSM 5.

**Table 1:** Demographics of the groups in the current study

	TD	ADHD	ASD−	ASD+	*F* value	Post hoc
*N*	29 (15 F)	23 (6 F)	15 (1 F)	18 (2 F)	–	–
Age	12.1 ± 1.4	12.5 ± 0.8	12.2 ± 1.1	12 ± 1	1.28	n.s
IQ	110.43 ± 15.55	103.19 ± 15.10	107.87 ± 19.56	98.84 ± 12.42	2.42	n.s
CBCL internalisation	52.60 ± 7.27	63.14 ± 10.52	69.43 ± 9.45	63.56 ± 7.66	14.73***	TD < ADHD,ASD−,ASD+
CBCL externalisation	47.20 ± 7.82	64.48 ± 11.19	60.80 ± 13.32	63.39 ± 8.35	16.74***	TD < ADHD,ASD−, ASD+
CBCL competence *T* score	62.67 ± 6.64	49.57 ± 7.55	48.73 ± 6.53	46.72 ± 9.30	23.86***	TD > ADHD,ASD−, ASD+
CBCL total *T* score	49.57 ± 6.31	66.38 ± 8.59	68.29 ± 9.98	66.11 ± 7.25	31.3***	TD < ADHD, ASD−, ASD+
SRS raw score	16.17 ± 11.44	59.05 ± 31.96	94.33 ± 39.04	88.47 ± 30.05	38.93***	TD < ADHD < ASD−, ASD+
SRS *T* score	44.83 ± 9.23	68.14 ± 9.34	77.13 ± 14.58	76.94 ± 7.49	54.23***	TD < ADHD < ASD−, ASD+
ADHD-FBB symptom score	–	1.26 ± 0.62	1.19 ± 0.52	1.44 ± 0.56	0.93	n.s
ADHD-FBB competence score	–	0.83 ± 0.49	1.32 ± 0.70	1.05 ± 0.51	3.41*	ADHD > ASD−
ADHD-SBB symptom score	–	0.91 ± 0.54	0.71 ± 0.47	1.20 ± 0.51	4.09*	ASD+ > ASD−
ADHD-SBB competence score	–	1.33 ± 0.77	1.77 ± 0.51	1.45 ± 0.65	1.96	n.s

Significance codes for *p* values: n.s. is *p* > 0.05, *p ≤ 0.05, **p ≤ 0.01, ***p ≤ 0.001. Higher symptom score and lower competence scores indicate increased symptoms

The correlation pattern within the different questionnaire scores, per group, has been shown in Table 3, and discussed in “Performance of visual search”.

### Apparatus

Stimuli were created in black and presented on a white background with dimensions of 1920 × 1080 pixels. Presentation^®^ software (Version 17.2, Neurobehavioral Systems, Inc., Berkley, CA, www.neurobs.com) was used for displaying the stimuli. Eye movements were recorded with the RED250 eye tracker (SensoMotoric Instruments GmbH) using a sampling rate of 120 Hz. Due to a technical mishap, the eye movements of four control participants, and five participants from clinical groups were recorded at a frequency of 60 Hz. The statistical analysis was carried out with and without these participants, showing that significance levels remained the same. BeGaze 3.7 (SensoMotoric Instruments GmbH) was used to define fixation as events with a minimum duration of 60 ms at a maximum dispersion of 2°, export event data, produce scanpaths and for exploratory analysis. R Software (version 3.4.3), packages provided by R (“basic”, “car”, “psych”, “dplyr”, “stats”, “ggplot2”, “lsr”), and R Studio (version 1.1.423) [47] were used for pre-processing of data, statistical analysis, and visualisations. MATLAB (R2016a) [36] was used to carry out the Recurrence Quantification Analysis [5] and the Multi-Match Model [13].

### Procedure

Participants sat approximately 70 cm in front of a screen in a sound-attenuated cabin, while their eye-movements were recorded. Each session was 60–90-min long and consisted of a battery of five tasks administered in a counterbalanced order across the participants of each group. The present article focuses on the Visual Search task. 5-point calibration and 4-point validation processes were successfully administered before collecting oculomotor data for the present task. The task started with standardised step-by-step instructions by the experimenter, followed by two un-timed trials alongside the experimenter and a ten-trial practice block with regular feedback from the experimenter, ensuring participants’ understanding. The practice block was succeeded by the main block of the task. To measure IQ, CFT-20 was administered in a second appointment as a group testing in groups of 3–6 participants.

### Task and stimuli

**Fig. 1:**
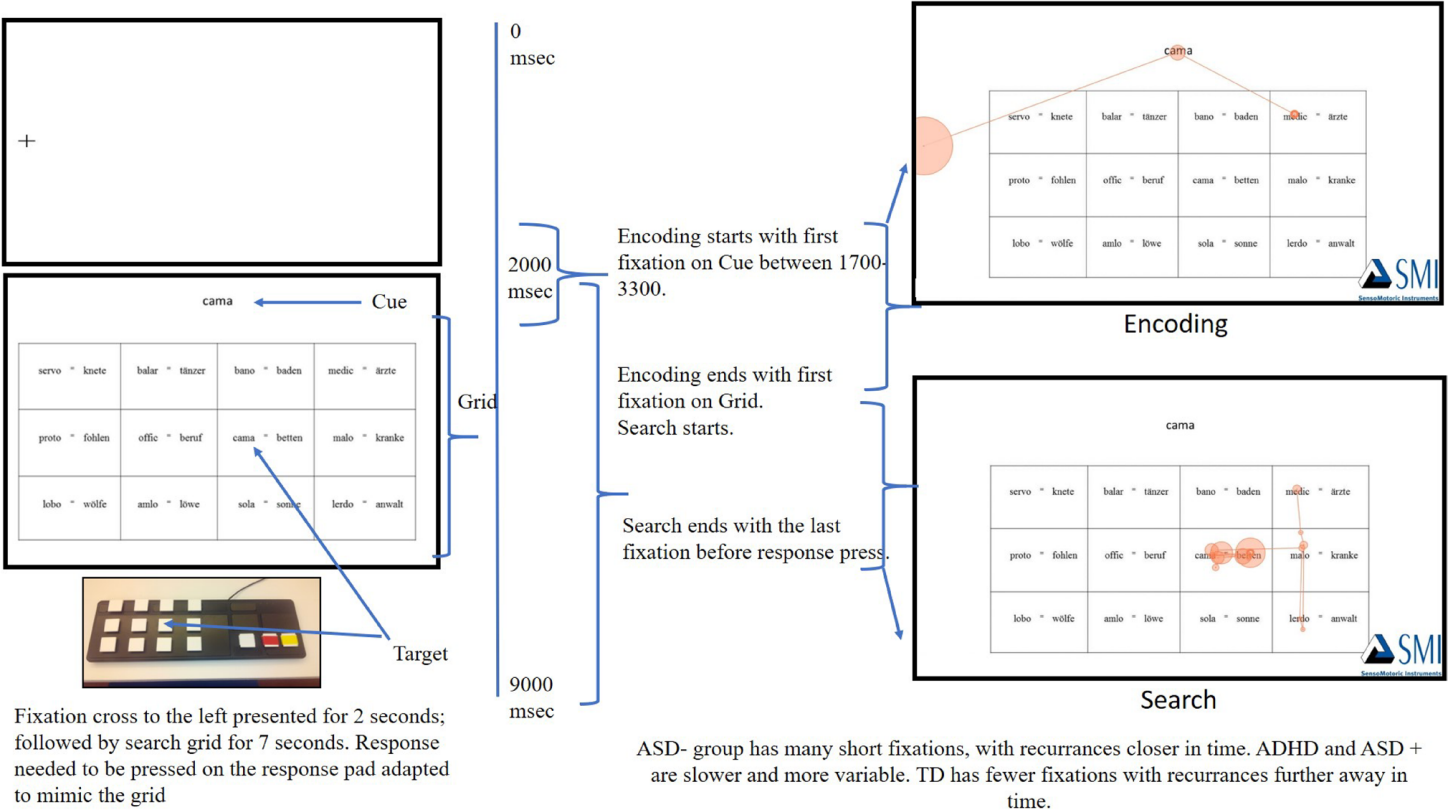
Example of a trial

The Visual Search Task entailed looking for a foreign language word as a cue word among a grid of foreign language words and their German “translations” (e.g., the word “cama” shown as cue word in Fig. 1, translates to “Betten” (“beds”) in German). At the beginning of each trial, a fixation cross was presented for 2 s at the left side of the screen, followed by the cue and grid for 7 s. Participants needed to respond on a response pad replicating the fields of the grid (see Fig. 1). The main block consisted of 30 trials and lasted 4.5 min. Each trial had a unique cue word. To control for exogenous sources of intra-subject variability, words in all 30 trials had been equated for length and number of syllables. Since 30 trials were administered across 12 target positions, 6 target positions were repeated twice across 12 trials and 6 target positions were repeated thrice across 18 trials.

### Analysis

Only trials with correct answers were included in the analysis. Corrected trials, i.e. trials where the first button response was incorrect but was successfully corrected with the following button press, were considered as correct trials. All time points below are with reference to the onset of the fixation cross on the left.

#### Segmentation

For segmenting, rectangular Areas of Interest (AOI) were marked for Cue, Grid, and Target. Each trial was broken down into two periods for analysis (shown in Fig. 1). The first was *Encoding and Initiation of Search*. The start of this segment was taken as the first fixation on Cue between 1700 and 3300 ms from trial onset. These cut-offs were selected based on a preliminary exploratory analysis to include the largest possible range of first fixations, including anticipatory and delayed fixations. If no fixation occurred directly into the AOI for Cue within this time frame, the trial was not analysed further. To prevent scanning of the grid before encoding of the cue, no more than one fixation lasting no more than 300 ms was allowed in the grid prior to fixation on cue. The end of the Encoding and Initiation of Search segment was taken as the first fixation on the grid after the first fixation on cue. The second segment was Search*,* defined as the phase from the first fixation on the grid after the first fixation on cue, until the last fixation on target, before the correct button response.

#### Dependent variables

For all variables, means were used as an indicator of performance on search and SD as a measure of intra-subject variability. Saville (2011) calculated the reliability of ISV using different measures. They found RTSD to be one of the best performing measures, and somewhat better than other measures such as CVRT. Further, the CVRT assumes a dependency of ISV on the mean which is not true in the case of increased ISV in ADHD [see, for instance, Feige et al. 2013; PLoS One, Vol. 8 (10)]. Hence, our decision is to use RTSD.

For the Encoding and Initiation of Search segment, the intra-subject mean and SD for the following four variables were analysed. Entry time to cue was calculated as the start time of the first fixation on the AOI ‘Cue’, relative to cue onset. Fixation duration and fixation count on cue were defined as total duration and number of fixations on AOI ‘Cue’ in the above-defined encoding phase. Entry time to grid was calculated as the start time of the first fixation on AOI ‘Grid’ after the first fixation on AOI ‘Cue’. For the Search Segment, all fixations were taken together to calculate fixation count and fixation duration per participant per trial. Further, two scan-path analysis models were carried out [8]. First, the Recurrence Quantification Analysis (RQA) of the search segment giving the variables Recurrence, Determinism, Laminarity, and Centre of Recurrence Mass was carried out on all trials of all participants. For RQA analysis, each cell in the grid was taken as an AOI, along with cue as another AOI. Recurrence is the percentage of fixations that were repeated per AOI. Determinism quantifies repeated scanpaths. Laminarity quantifies detailed exploration of AOIs. Centre of Recurrence Mass (CORM) quantifies the temporal structure of recurrences made. Further details can be found in Anderson et al. [13]. In a follow-up analysis directed explicitly towards ISV of recurrence, the variables were re-calculated per cell, where targets present in the same cell were subtracted from each other. The second scan path analysis carried out used Multi-Match [13], which measures the similarity of two scanpaths and gives the variables Vector Similarity, Length Similarity, Direction Similarity, Position Similarity, and Duration Similarity. Multi-Match analysis was directed at calculating ISV. The scan paths of trials per participant, where targets were present in the same cell position, were compared. Finally, per participant, the mean reaction times (RT) and SD of reaction times (RTSD) were calculated as the time of motor response from the stimulus onset.

#### Statistical analysis

For each of the dependent variables described above, analysis of variance (ANOVA) was carried out with GROUP as a between-subject factor with four levels—TD, ADHD, ASD− and ASD+. All post hoc analysis was calculated by the Tukey’s test. Including IQ as a covariate had only marginal effects on the main effect of GROUP; therefore, only the results without the covariate IQ are reported here.

## Results

**Fig. 2:**
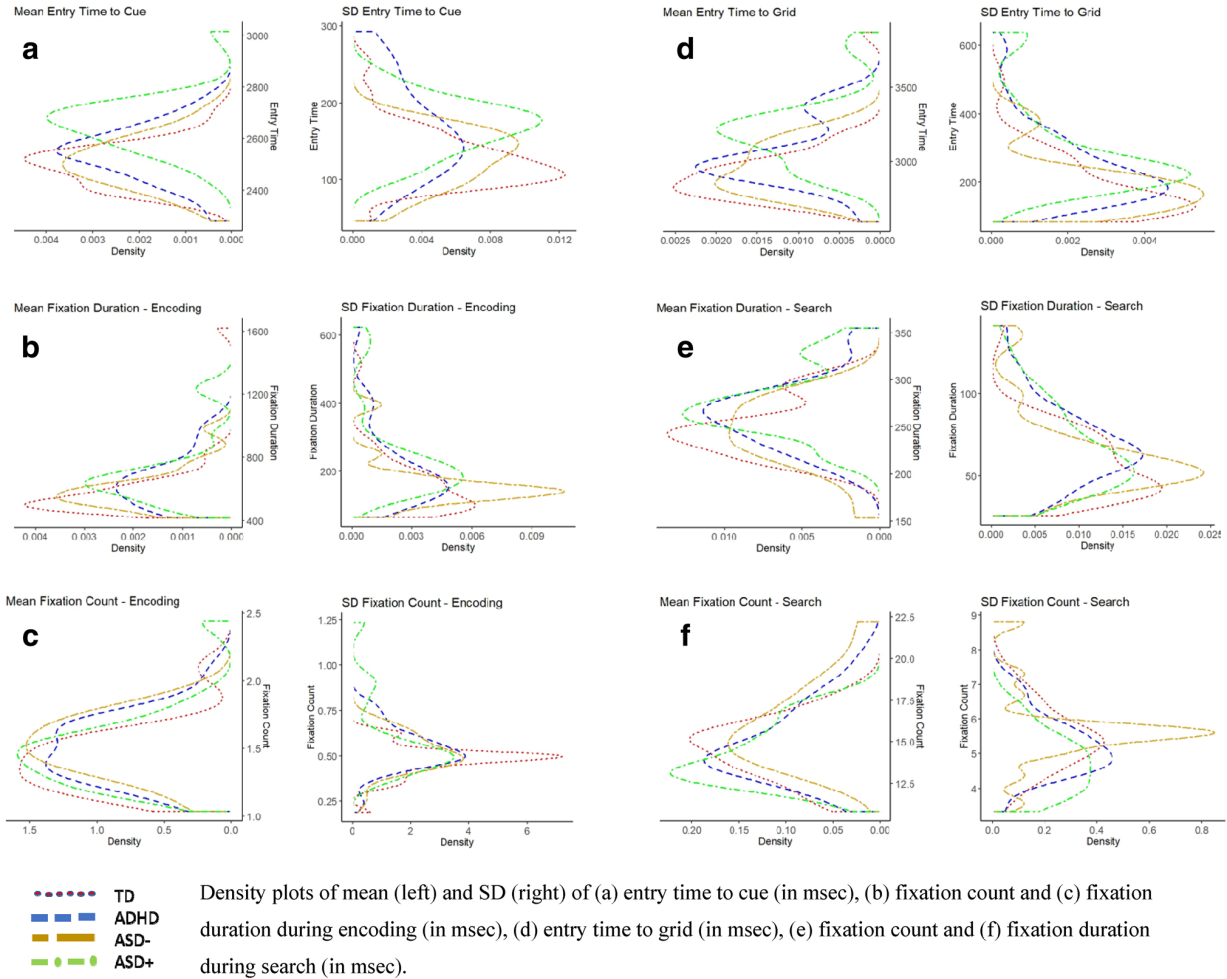
Density plots of mean and SD of encoding, initiation of search and search variables

Table 2 provides the descriptive statistics of variables calculated for encoding, initiation of search, search segments and behavioural outcomes. Figure 2 provides density plots for entry time to cue, fixation count and duration during encoding, entry time to grid and fixation count and duration during search. The sub-sections below discuss (1) the performance and (2) the ISV of the visual search task in the four groups.

**Table 2:** Descriptive statistics and significant between-group differences of all the variables used to quantify encoding, initiation of search and search

Variable	TD	ADHD	ASD−	ASD+	Significant Post hocs
Total number of valid trials	780	565	393	401	–
Mean number of valid trials	26.9 ± 2.83	24.57 ± 5.24	26.2 ± 2.76	23.06 ± 5.01	ASD+ < TD
*Performance on visual search task*					
Encoding and initiation of search					
Mean entry to cue (ms)	2493.01 ± 82.81	2540.47 ± 110.56	2508.11 ± 98.86	2662.29 ± 120.31	TD, ASD−,ADHD < ASD+
Mean fixation duration (ms)	604.29 ± 219.72	655.08 ± 168.87	600.59 ± 144.55	716.89 ± 220.02	–
Mean fixation count	1.42 ± 0.25	1.5 ± 0.24	1.52 ± 0.22	1.51 ± 0.3	–
Mean entry to grid (ms)	2921.3 ± 227.87	3045 ± 220.74	2957.09 ± 166.65	3208.27 ± 260.43	TD, ASD− < ASD+
Search					
Mean fixation duration (ms)	248.6 ± 30.68	263.96 ± 36.48	241.98 ± 37.12	276.5 ± 38.01	TD, ASD− < ASD+
Mean fixation count	14.52 ± 1.86	14.9 ± 2.42	16.17 ± 2.53	14.29 ± 1.76	TD, ASD+ < ASD−
Mean recurrence	24.27 ± 4.41	24.75 ± 3.65	24.38 ± 3.4	21.73 ± 6.25	–
Mean determinism	38.95 ± 4.25	38.78 ± 5.87	39.34 ± 5.06	37.32 ± 7.63	–
Mean laminarity	35.08 ± 5.69	35.66 ± 6.2	36.55 ± 5.87	32.36 ± 8.27	–
Mean CORM	38.43 ± 2.08	38.3 ± 2.09	35.58 ± 3.49	38.23 ± 2.26	ASD− < ASD+ , ADHD, TD
Reaction time and accuracy					
Mean reaction time (ms)	5784.86 ± 680.25	6218.72 ± 660.87	6107.88 ± 542.12	6698.39 ± 545.34	TD, ASD− < ASD+
Inaccurate trials	0.20 ± 0.48	0.34 ± 0.77	0.46 ± 0.83	0.21 ± 0.41	TD < ASD+
Missing trials	0.6 ± 1.13	1.39 ± 1.46	1.33 ± 1.45	2.89 ± 2.68	TD < ASD+
*Intra subject variability*					
Encoding and initiation of search					
SD entry time to cue (ms)	128.57 ± 41.22	162.58 ± 60.51	126.89 ± 36.39	169.07 ± 35.03	TD, ASD− < ASD+, TD < ADHD
SD fixation duration (ms)	158.06 ± 91.03	219.63 ± 128	161.45 ± 77.49	246.45 ± 137.96	TD < ASD+
SD fixation count	0.47 ± 0.09	0.54 ± 0.12	0.51 ± 0.11	0.59 ± 0.23	TD < ASD+
SD entry time to grid (ms)	182.14 ± 92.74	245.62 ± 111.49	191.9 ± 88.1	295.48 ± 138.34	TD, ASD− < ASD+
Search					
SD fixation duration (ms)	59.92 ± 24.74	69.82 ± 26.22	61.78 ± 26.82	66.92 ± 24.38	–
SD fixation count	5.38 ± 0.86	5.24 ± 0.88	5.5 ± 0.9	4.72 ± 0.87	–
SD recurrence	20.62 ± 6.25	19.94 ± 4.72	18.88 ± 5.1	17.91 ± 6.28	–
SD determinism	21.23 ± 3.78	21.86 ± 4.02	21.11 ± 2.97	20.79 ± 6.56	–
SD laminarity	20.43 ± 3.17	20.68 ± 4.26	20.1 ± 3.01	19.93 ± 5.61	–
SD CORM	7.9 ± 2.23	7.63 ± 2.39	7.63 ± 2.19	7.77 ± 2.32	–
Reaction time					
SD reaction time (ms)	1194.97 ± 204.72	1280.23 ± 249.18	1365.66 ± 213.47	1423.18 ± 180.68	TD < ASD+

Per group and variable mean ± SD have been reported with the start of trial taken as baseline 0. The significant post hoc column shows the significant between-group differences on the calculated ANOVAs

### Performance of visual search

#### Encoding and Initiation of search

For mean entry time to cue, the effect of GROUP (*F*_(3,81)_ = 11.10, *p* < 0.00001, *η*^2^ = 0.29) showed the ASD+ group to be the slowest and significantly different from the TD (*t*_(81)_ = 5.52 *p* < 0.001, *d* = 1.22), ADHD (*t*_(81)_ = 3.79, *p* < 0.005, *d* = 0.84) and ASD− (*t*_(81)_ = 4.32, *p* < 0.001, *d* = 0.96) groups, which did not differ from each other. No significant GROUP differences were observed for mean fixation duration on cue (*F*_(3,81)_ = 1.36, *p* = 0.25, *η*^2^ = 0.04) and mean fixation count on cue (*F*_(3,81)_ = 0.82, *p* = 0.48, *η*^2^ = 0.02). For the effect of GROUP on mean entry time to grid (*F*_(3,81)_ = 6.60, *p* < 0.0005, *η*^2^ = 0.19) the ASD+ group was again found to be significantly slower than the TD (*t*_(81)_ = 4.26, *p* < 0.0005, *d* = 0.94) and ASD− (*t*_(81)_ = 3.20, *p* = 0.01, *d* = 0.71) groups, but not the ADHD group, which performed comparable to the TD group.

#### Search

##### Fixation duration and count in search

The TD and ASD− groups had shorter mean fixation duration during search as compared to the ADHD and ASD+ groups. The GROUP effect (*F*_(3,81)_ = 3.62, *p* < 0.05, *η*^2^ = 0.11) and post hoc tests showed significantly longer fixation durations only in ASD+, not ADHD group, compared to TD (*t*_(81)_ = 2.65, *p* < 0.05, *d* = 0.58) and ASD− (*t*_(81)_ = 2.81, *p* < 0.05, *d* = 0.62) participants.

The ANOVA measuring GROUP effects on fixation count during search was marginally significant (*F*_(3,81)_ = 2.71, *p* = 0.05, *η*^2^ = 0.09) and post hoc tests showed ASD− group to have higher number of fixations than ASD+ (*t*_(81)_ = 2.59, *p* = 0.05, *d* = 0.57) and TD groups (*t*_(81)_ = 2.50, *p* = 0.06, *d* = 0.55), but did not differ significantly from ADHD at post hoc tests.

##### RQA values in search

From the RQA values, mean CORM values revealed a significant effect of GROUP (*F*_(3,81)_ = 5.36, *p* < 0.005, *η*^2^ = 0.16), with the ASD− group showing significantly lower CORM values than TD (*t*_(81)_ = 3.70, *p* < 0.005, *d* = 0.81), ADHD (*t*_(81)_ = 3.38, *p* < 0.005, *d* = 0.75) and ASD+ (*t*_(81)_ = 3.12, *p* = 0.01, *d* = 0.69) groups^1^. This means that the ASD− group was faster at recognising a word to be re-inspected. Mean recurrence (*F*_(3,81)_ = 1.78, *p* = 0.15, *η*^2^ = 0.06), mean determinism (*F*_(3,81)_ = 0.33, *p* = 0.79, *η*^2^ = 0.01), mean laminarity (*F*_(3,81)_ = 1.45, *p* = 0.23, *η*^2^ = 0.05) did not show significant GROUP effects.

#### Behavioural outcomes

An overall GROUP effect on mean RT (*F*_(3,81)_ = 8.01, *p* < 0.00005, *η*^2^ = 0.22) revealed that the ASD+ group was significantly slower than the TD group (*t*_(81)_ = 4.86, *p* < 0.001, *d* = 1.08), and the ASD− group (*t*_(81)_ = 2.69, *p* < 0.05, *d* = 0.59). Incorrect responses were very rare across groups (0.27 ± 0.62), and did not differentiate between them. However, group differences were observed for missing responses after removing two outliers from the ASD + group (defined as values larger than 3 SDs within this group) (*F*_(3,79)_ = 5.39, *p* = 0.001, *η*^2^ = 0.17), with the ASD+ group having significantly more (*t*_(79)_ = 3.97, *p* < 0.001, *η*^2^ = 0.89) missing responses than the TD group.

*To summarise the performance of visual search *The ASD− group, compared to the other three groups, exhibited short fixations, with recurrences or revisits closer in time during search. Also, the ASD+ and ADHD groups were both significantly slower than the TD and ASD− group as indicated by variables for encoding, initiation of search, search, and reaction time. Thus, the pattern indicative of the ASD− group is significantly different from the other groups in crucial ways.

### ISV of visual search

#### Encoding and initiation of search

For SD values, all the encoding and initiation of search variables showed significant GROUP effects, with the ADHD and ASD+ groups being more variable than the TD and ASD− groups. For SD entry time to cue (*F*_(3,81)_ = 4.86, *p* < 0.005, *η*^2^ = 0.15) and SD entry time to grid (*F*_(3,81)_ = 4.80, *p* < 0.005, *η*^2^ = 0.15), the ASD+ group was found to be significantly more variable than the TD (*t*_(81)_ = 2.96, *p* < 0.05, *d* = 0.65 and *t*_(81)_ = 3.49, *p* < 0.005, *d* = 0.77, respectively) and ASD− (*t*_(81)_ = 2.65, *p* < 0.05, *d* = 0.58 and *t*_(81)_ = 2.73, *p* < 0.05, *d* = 0.60, respectively) groups; and the ADHD group was further found to be significantly more variable than the TD group (*t*_(81)_ = 2.67, *p* < 0.05, *d* = 0.59) for SD entry time to cue. For SD fixation duration (*F*_(3,81)_ = 3.18, *p* < 0.05, *η*^2^ = 0.10) and SD fixation count (*F*_(3,81)_ = 2.77, *p* < 0.05, *η*^2^ = 0.09) on cue, only the ASD+ group, not the ADHD group, was found to be more variable than the TD group (*t*_(81)_ = 2.65, *p* < 0.05, *d* = 0.58 and *t*_(81)_ = 2.80, *p* < 0.05, *d* = 0.62, respectively). However, SD of fixation duration on cue and fixation count on cue was no longer significant in the ANCOVAs recalculated with IQ as a covariate.

#### Search

##### Fixation duration and count in search

For SD of fixation duration for Search, the descriptive statistics were observed to be in the same direction, but these were not significant (*F*_(3,81)_ = 1.05, *p* = 0.37, *η*^2^ = 0.03). For SD fixation count for search, a similar pattern was observed. These findings were significant (*F*_(3,79)_ = 2.74, *p* = 0.05, *η*^2^ = 0.09), even though 2 outliers had to be removed from the TD and ASD− group, respectively. However, no differences were observed in the post hoc tests.

All group differences for all the RQA values, RQA subtracted variables, and multi-match model variables were not significant and revealed F-values of less than 1.

#### Behavioural outcomes

For RTSD (*F*_(3,81)_ = 4.81, *p* < 0.005, *η*^2^ = 0.15), only the ASD+ group, not the ADHD group, was significantly more variable than the TD group (*t*_(81)_ = 3.54, *p* < 0.005, *d* = 0.78).

*To summarise ISV of visual search *Although the ADHD group was consistently more variable than the TD group, this was not always found to be significant. The ASD+ group behaved very similarly to the ADHD group. In all four encoding and initiation of search variables as well as reaction time, the ASD+ group was even more variable than the ADHD group, making it consistently and significantly different from the TD group. The ASD+ group was also significantly different from the ASD− group with regard to entry time to cue and grid.

## Discussion

The primary aim of this study was to investigate the potential commonalities or dissociations between ADHD and ASD using behavioural and oculomotor parameters of a visual search task in three clinical groups, namely ADHD, ASD− and ASD+ , along with a TD control group. This goal was achieved by studying potential endophenotypes of ASD (i.e., visual search performance) and ADHD (i.e., ISV). The present study found evidence supporting a double dissociation between ASD and ADHD on the constructs of visual search performance and ISV.

### Performance of visual search

The ASD− group showed clear signs of better visual search performance—this group was either at par with the TD group or performed better as indicated by the CORM values. This is in line with most of the literature [25, 27, 31, 43] showing improved search performance as a sign for heightened local processing [11, 39] in ASD. However, the ASD  group did not show the same pattern as its ‘pure’ ASD counterpart. While ASD  search could be characterised as a fast, efficient search with consistently shorter fixations closer in time, the ASD+ group was particularly slow, inefficient, and had significantly longer fixations. The ASD− group presented superior performance on search. The ASD+ group not only presented an absence of the superior search found in ASD− (as the ADHD group did), but rather tended to perform particularly poorly at the task.

The ASD+ group had greater ASD and ADHD symptoms than either of the ASD− and ADHD groups individually (Table 1). Further, these symptoms were moderately and positively correlated in the ASD+ group (Table 3). At first glance then, it may seem that the ASD+ group is at a disadvantage because of inflated and increased pathology. However, this explanation may be too simplistic. Unterrainer et al. [54], in a cross-sectional study, have shown a steeper increase with age for performance on planning in the ASD+ , in comparison to the ASD− group. They found support for task rigidity in ASD− as evidenced by longer planning durations, and argue that the ADHD symptomatology in ASD+ enables planning by disrupting this rigid focus normally associated with ASD. There has also been some discussion of ASD+ being an addition of ASD− and ADHD characteristics [47, 52, 53]. However, the pertinent results have not always been conclusive. For example, Tye et al. [52] found inhibitory processing in ASD+ to be an additive outcome of ADHD and ASD, but response preparation to be non-additive. Adding to the complexity is the fact that while deficits have been studied, however scantily, how these increased deficits interact with possible strengths has not yet been addressed. Visual search is a strength in ASD that seems to dissipate completely with the additional presence of ADHD symptoms. This is markedly different than additive or non-additive deficits of each disorder.

**Table 3:** Correlations between ADHD symptoms, ASD symptoms, internalisation and externalisation

Correlations between	ADHD	ASD−	ASD+
SBB–SRS	0.53	− 0.08	0.60
SBB–internalisation	0.33	− 0.30	0.17
SBB–externalisation	0.34	− 0.77	0.02
SRS–internalisation	0.39	0.66	0.36
SRS–externalisation	0.64	0.83	0.43
FBB–SRS	0.81	0.51	0.80
FBB–internalization	0.18	0.06	0.19
FBB–externalization	0.66	0.56	0.32

Based on the above evidence, looking at ASD+ as having a different constellation than ASD− seems reasonable. While the behavioural phenotype of ASD+ may look like the addition of two disorders, this assumption cannot be extended to the underlying cognitive processes studied here. This is an important distinction to make while entertaining the possibility that potential endophenotypes of ASD− , such as better local processing, may not be the same as potential endophenotypes of ASD+ .

The present study found no evidence for better performance on visual search or heightened attention to local elements of stimuli in the ADHD group. The present study also did not find indications of worse performance in the ADHD groups; this was expected. The review from Mullane and Klein [40] showed that participants with ADHD, in visual search tasks, show disrupted performance only in the easiest and most difficult conditions. The present task enabled a good accuracy rate, probably owing to ceiling effects (Table 2), indicating it was not too difficult; and based on the authors’ pilot tests with adults, who found the task engaging and not particularly easy, it can be assumed that the present task involving the search for a foreign word from 23 distractor words is not easy for 10–13-year olds either. Thus, the parameters for performance of search would not, presumably, be much worse in the ADHD group, as was observed on all performance of search parameters (Table 4).

**Table 4:** Post hoc (Tukey) *t* values for all main variables

Variable		ADHD	ASD−	ASD+		ADHD	ASD−	ASD+
	Performance on visual search (mean)				Intra subject variability (SD)			
Encoding and initiation of search								
Entry time to cue	TD	1.67	0.46	5.52***	TD	2.67*	0.11	2.97*
	ADHD		0.95	3.79**	ADHD		2.36	0.45
	ASD−			4.32***	ASD−			2.65*
Fixation duration	TD	0.99	0.007	1.84	TD	1.98	0.09	2.65*
	ADHD		0.83	0.86	ADHD		1.58	0.77
	ASD−			1.57	ASD−			2.19
Fixation count	TD	1.22	1.29	1.08	TD	1.67	0.83	2.80*
	ADHD		0.21	0.05	ADHD		0.60	1.19
	ASD−			0.25	ASD−			1.65
Entry time to grid	TD	1.98	0.50	4.26***	TD	2.10	0.28	3.49**
	ADHD		1.18	2.30	ADHD		1.49	1.46
	ASD−			3.20*	ASD−			2.73*
Search								
Fixation duration	TD	1.56	0.59	2.63*	TD	1.67	0.45	1.16
	ADHD		1.89	1.13	ADHD		0.97	0.38
	ASD−			2.81*	ASD−			0.57
Fixation count	TD	0.65	2.51	0.36	TD	0.51	0.41	2.49
	ADHD		1.85	0.92	ADHD		0.88	1.87
	ASD−			2.59	ASD−			2.49
Recurrence	TD	0.37	0.73	1.88	TD	0.43	0.96	1.62
	ADHD		0.24	2.12	ADHD		0.55	1.14
	ASD−			1.68	ASD−			0.49
Determinism	TD	0.03	0.28	0.79	TD	0.51	0.08	0.33
	ADHD		0.29	0.73	ADHD		0.51	0.77
	ASD−			0.93	ASD−			0.20
Laminarity	TD	0.40	0.78	1.40	TD	0.22	0.25	0.41
	ADHD		0.41	1.69	ADHD		0.42	0.59
	ASD−			1.91	ASD−			0.12
CORM	TD	0.19	3.70**	0.27	TD	0.42	0.37	0.18
	ADHD		3.38**	0.09	ADHD		0.005	0.20
	ASD−			3.12*	ASD−			0.18
Reaction time								
Reaction time	TD	2.43	1.28	4.49***	TD	1.42	2.50	3.54**
	ADHD		0.85	2.05	ADHD		1.19	2.11
	ASD−			2.70*	ASD−			0.77

Although still within three standard deviations, one of the participants from the ASD− group had a CORM value under 30, making it seem further away from the rest of the scores. Results were therefore recalculated without this participant. A significant group effect was still observed (*F*(3,82) = 3.55, *p* < 0.01, *η*^2^ = 0.11) with the ASD− group showing significantly lower CORM values than TD (diff = 2.14, *p* < 0.01), and ADHD (diff = 2.04, *p* < 0.05) groups. Group differences between ASD+ and ASD− were no longer significant, but the trend remained (diff = 1.90, *p* = 0.06)

Significance codes for *p* values: *p ≤ 0.05, **p ≤ 0.01, ***p ≤ 0.001

### ISV of visual search

While increased ISV was observed for all encoding and initiation of search variables in the ASD+ group, the ADHD group manifested increased ISV only for entry time to cue. Although, descriptively, the ADHD group was consistently more variable than the TD group, this was not always found to be significant. This was a surprising finding considering ISV is the most consistent finding in the ADHD literature [28, 33–35, 48], and is, therefore, discussed below.

**Fig. 3:**
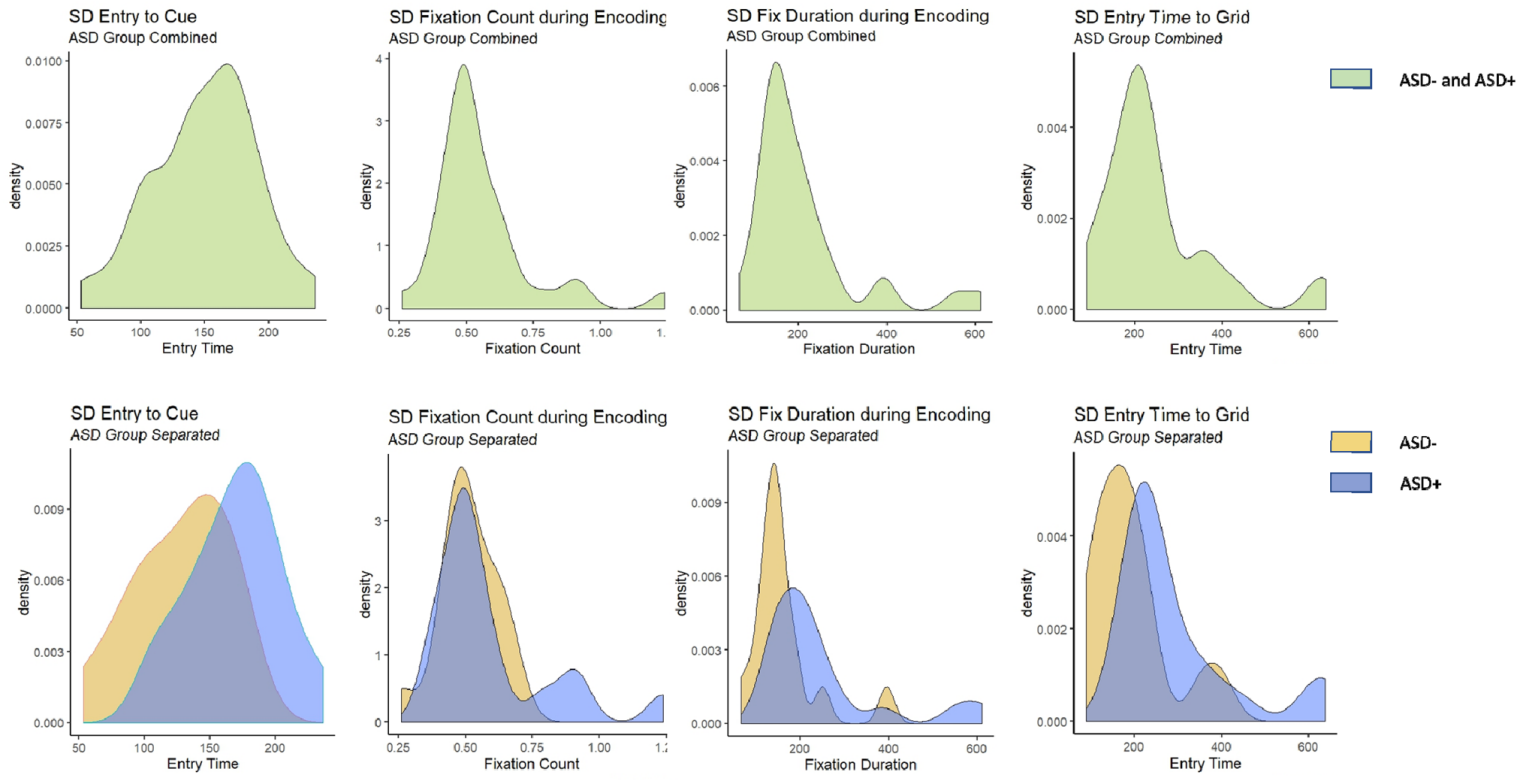
Histograms of ISV variables—with ASD+ and ASD− as combined or separate groups. Histograms (from left to right) of SD entry time to cue (in msec), SD fixation count during encoding, SD fixation duration during encoding (in msec) and SD entry time to grid (in msec)

Studies that investigate ISV in ASD in comparison to an ADHD group, without separating participants with and without ADHD, have had mixed findings [18, 54]. However, studies that separated ASD− and ASD+ groups found increased variability only in the ASD+ groups [48, 52, 53]. To further understand if this ASD sub-group distinction was the reason the present study also showed increased ISV in only the ASD+ group, the ISV variables for encoding and initiation of search were re-analysed with ASD as one group comprising of both ASD+ and ASD− patients. As expected, in this combined group no significant differences between ASD and the other groups were observed. Figure 3 shows histograms with ASD+ and ASD− treated as one group above and as two separate groups below. The consistent observations that ISV is increased in ASD, only when ADHD symptoms are present, can be explained by looking at ISV as not just a candidate endophenotype of ADHD but also a trans-diagnostic phenotype [28], associated with symptom domains such as attention-deficit and hyperactivity that can cut across several disorders including ASD.

Looking at ISV as a trans-diagnostic phenotype explains why it is observed in ASD+ , but not why ASD+ shows greater ISV than the ADHD group itself. Further, ISV increased only at entry time to cue for the ADHD group, but on all encoding and initiation of search variables for the ASD+ group. A possible explanation is that increased ISV in ADHD and ASD+ point to different kinds of neuronal variability. Dinstein et al. explain how neuronal variability can have different sources and these can be deconstructed in several ways; for instance, evoked in early versus late parts of a trial, stimulus or response-locked, localised to a specific brain region or spread across the brain. When taken in a context of clinical disorders, increased neuronal variability, although not specific to one particular disorder, may show distinct forms in different disorders. For instance, resting state neuronal variability can be observed in schizophrenia and variability in response-locked P300 brain potentials is observed in ADHD [14, 49, 57]. It is, therefore, possible, that although increased ISV is a trans-diagnostic phenotype observable in autism, the underlying neuronal variability in ADHD and ASD+ differs. This may manifest as increased ISV for ADHD as an oculomotor response to the stimulus onset; manifesting in increased ISV at the start of the task, as measured by entry time to cue. Increased ISV in ASD+ , however, manifests throughout encoding up until search is initiated. Neither ADHD nor ASD+ seems to be more variable than TD during the search task.

## Conclusions, implications, limitations and future directions

The present study found support for a double dissociation between pure ASD− and ADHD groups on the constructs of local processing and ISV. It has also revealed that the ASD+ group may be seen as a separate group, with its own range and intensity of symptoms and traits that are reflected in a distinct pattern of performance, rather than a mere addition of ASD and ADHD. With respect to implications, we re-iterate that the present study adds to the recently growing literature on the potential overlaps between ADHD and ASD. Since the two ‘pure’ clinical groups could not be compared to the co-morbid group before DSM 5, studies until that point may have ambiguous diagnoses in their clinical groups, which the present study has successfully differentiated and investigated. The present study had limitations in terms of its relatively modest sample size, requiring replication in larger samples. Further, there may be interacting and confounding variables that the present study has not considered, such as reading competencies of each participant. Replications need to collect reading scores from participants as controls. It is also possible that certain stimuli (such as words or pictures for superheroes or video games) are more interesting to participants and influence quality of search. Future studies could try different stimuli to see if these lead to different moderator effects. The present study also used an array of tasks culminating in a 60–90-period testing session. Future studies could try and reduce the total testing time and/or include more breaks to investigate if this improves the performace of the ASD+ group in relation to the ADHD and ASD− groups. Finally, different age groups need to be investigated to understand if group differences in search strategies change with age. Future research needs to continue testing different ASD and ADHD constructs in the three clinical groups so that a comprehensive overview can be formed about what constructs are typical to ASD− and ADHD, what constructs add up in the ASD+ group and which are the constructs that need a more complex interaction model. While some research [7, 48, 52, 53], including the current paper, has done this with motor movements, executive function deficits, social and emotion processing and ISV, they remain to be replicated. Other constructs such as global processing and language deficits remain to be studied. ASD and ADHD both manifest considerable amounts of phenotypical variability [6, 18, 23, 44]. Identifying and extensively describing potential subgroups can help clinicians make an informed diagnosis and provide individually tailored therapy.
